# High genetic variability of Schmallenberg virus M-segment leads to efficient immune escape from neutralizing antibodies

**DOI:** 10.1371/journal.ppat.1009247

**Published:** 2021-01-26

**Authors:** Kerstin Wernike, Ilona Reimann, Ashley C. Banyard, Franziska Kraatz, S. Anna La Rocca, Bernd Hoffmann, Sarah McGowan, Silke Hechinger, Bhudipa Choudhury, Andrea Aebischer, Falko Steinbach, Martin Beer

**Affiliations:** 1 Institute of Diagnostic Virology, Friedrich-Loeffler-Institut, Greifswald—Insel Riems, Germany; 2 Department of Virology, Animal and Plant Health Agency Weybridge, Addlestone, United Kingdom; 3 Department of Experimental Animal Facilities and Biorisk Management, Friedrich-Loeffler-Institut, Greifswald—Insel Riems, Germany; 4 School of Veterinary Medicine, Faculty of Health and Medical Sciences, University of Surrey, Guildford, United Kingdom; University of Glasgow, UNITED KINGDOM

## Abstract

Schmallenberg virus (SBV) is the cause of severe fetal malformations when immunologically naïve pregnant ruminants are infected. In those malformed fetuses, a “hot-spot”-region of high genetic variability within the N-terminal region of the viral envelope protein Gc has been observed previously, and this region co-localizes with a known key immunogenic domain. We studied a series of M-segments of those SBV variants from malformed fetuses with point mutations, insertions or large in-frame deletions of up to 612 nucleotides. Furthermore, a unique cell-culture isolate from a malformed fetus with large in-frame deletions within the M-segment was analyzed.

Each Gc-protein with amino acid deletions within the “hot spot” of mutations failed to react with any neutralizing anti-SBV monoclonal antibodies or a domain specific antiserum. In addition, *in vitro* virus replication of the natural deletion variant could not be markedly reduced by neutralizing monoclonal antibodies or antisera from the field. The large-deletion variant of SBV that could be isolated in cell culture was highly attenuated with an impaired *in vivo* replication following the inoculation of sheep.

In conclusion, the observed amino acid sequence mutations within the N-terminal main immunogenic domain of glycoprotein Gc result in an efficient immune evasion from neutralizing antibodies in the special environment of a developing fetus. These SBV-variants were never detected as circulating viruses, and therefore should be considered to be dead-end virus variants, which are not able to spread further. The observations described here may be transferred to other orthobunyaviruses, particularly those of the Simbu serogroup that have been shown to infect fetuses. Importantly, such mutant strains should not be included in attempts to trace the spatial-temporal evolution of orthobunyaviruses in molecular-epidemiolocal approaches during outbreak investigations.

## Author summary

Schmallenberg virus (SBV) is a pathogen of veterinary importance and is used as a model virus for studying peribunyaviruses, a complex and highly divergent family of RNA viruses. An SBV-infection of naïve dams during pregnancy may lead to the induction of severe malformation in the fetus. In the medium (M) genomic segment of SBV and related viruses, a region of high sequence variability was detected, affecting the N-terminal major immunogenic domain of envelope protein Gc in malformed fetuses. Variation of this mutation hot spot in fetuses was demonstrated to result in immune-evasion from neutralizing activity in the infected fetus. Accordingly, the neutralization capacity of SBV-specific antisera collected from SBV-infected animals on the *in vitro* growth of such a variant virus isolate was severely impaired. Furthermore, the deletion mutant was attenuated for adult sheep. This study provides important new insights into the mechanisms of virus persistence within chronically infected malformed fetuses and explains the mystery of the “hot-spot” of sequence variations not only observed in SBV, but also in related viruses. The model proposed can represent an example of virus mutations resulting in antibody induced immune escape.

## Introduction

Schmallenberg virus (SBV), which emerged in 2011 in Central Europe, is transmitted by Culicoides biting midges and causes no or only mild non-specific and short-lived clinical signs in adult ruminants [1], but can induce premature birth, stillbirth, or severe malformations in the offspring when immunologically naïve animals are infected during a vulnerable period of pregnancy [2]. SBV is the first European member of the Simbu serogroup of orthobunyaviruses detected; further Simbu viruses such as Akabane virus (AKAV) or Aino virus are widely distributed in Asia, Africa, and Oceania and play an important role in animal health in those countries [3].

Like other orthobunyaviruses, Schmallenberg virions contain three segments of negative-stranded RNA genome. The large (L) genome segment encodes the RNA-dependent RNA polymerase (RdRp); the medium (M) segment encodes the viral glycoproteins Gn and Gc, as well as a non-structural protein (NSm); and the small (S) segment the nucleocapsid protein (N) and the non-structural protein NSs [4–6].

Within the insect vector season, in which the virus was detected for the first time (year 2011), SBV spread very rapidly across the European ruminant population. In the center of the epizootic in northwestern Germany, the Netherlands, and Belgium more than 90% of the tested cattle were seropositive. During the following years, SBV circulated further in Germany, albeit at a much lower level. However, in summer and autumn 2014, the virus circulated again widely in continental Europe [7] and in the following winter the frequency of offspring displaying SBV-induced malformation increased. In 2015, and the following winter, SBV cases were again reported only sporadically. However, in 2016 and in 2019 the virus circulated again on a larger scale in the European ruminant population and in the following winters (2016/17 and 2019/2020) an increasing number of malformed calves and lambs were born [8–11].

In malformed calves and lambs, very high SBV genome copy numbers have been demonstrated, mainly in the central nervous system (CNS) [12,13], and in such cases a high genetic variability was observed within the M-segment [10,14,15]. Interestingly, this “hot-spot” region lies within the 5’-terminal part of the M-segment, encoding for a domain (=“head domain”) within the major SBV envelope protein Gc recently shown to be highly immunogenic and crucial for neutralization [16–18]. For Bunyamwera virus, the prototype orthobunyavirus, the N-terminal half of the Gc protein was shown to be dispensable for virus replication in cell culture [19], but deletion mutants showed impaired growth characteristics [20]. Also for Maguari orthobunyavirus it could be demonstrated that following passage in the presence of 5-fluorouracil the N-terminal domain of Gc is not required for replication in cell culture [21]. In the case of SBV, the observed variability is in distinct contrast to the otherwise reported genome stability of circulating SBV detected in acutely infected ruminants [7,10,11,22]. Here, we suggest that fetal infection provides an environment that favors deleterious viral mutations that enables immune escape from neutralizing antibodies, but limits the ability of the virus to further replicate and spread.

## Results and discussion

### SBV Gc deletion mutant viruses occur naturally

In January 2015, different tissue samples (brain, lung, placenta, and two unidentifiable organ samples) of a malformed lamb were submitted to the Friedrich-Loeffler-Institut to be analyzed for the presence of SBV-genome (submission number BH04/15). For the detection of viral RNA, independent real-time RT-PCR assays targeting either on the S-, the M-, or the L-segment [23] were used. By the S- and L-segment based assays, SBV genome could be detected in every sample with the lowest Cq value in the brain (Table 1).

**Table 1:** Real-time RT-PCR results of ovine tissue samples that were submitted to the Friedrich-Loeffler-Institut to be analyzed for the presence of SBV-genomes (submission number BH04/15). The brain, lung, placenta and two unidentified organ samples obtained from a malformed lamb were investigated by three different real-time PCR systems either based on the viral S-, M- or L-segment. Cq–quantification cycle.

Sample material	S-segment	M-segment	L-segment
Brain	16.9	No Cq	20.7
Organ sample 1	17.6	No Cq	20.9
Lung	34.0	35.6	34.9
Placenta	31.1	No Cq	33.5
Organ sample 2	25.2	No Cq	28.1

Surprisingly, using the M-segment-based PCR, only the lung sample was positive, every other tissue sample tested negative indicating nucleotide variations in the primer and/or probe binding sites or the presence of an M-segment of a related Simbu serogroup virus instead of an SBV-M. As in other viruses with segmented genomes, natural genetic reassortment occurs also in orthobunyaviruses [24–27]. Sequence analysis of the complete coding region of the M-segment was performed from the samples with the lowest Cq value in the S-segment based RT-PCR (brain and an unidentified organ sample) to determine reasons for PCR negativity. The sequences obtained from both analyzed samples were identical. In both cases, a large in-frame deletion of 414 nucleotides (nt) within the glycoprotein Gc coding region was detected. This demonstrated why PCR of the M segment had failed. No sequences could be generated from the lung sample, likely because of low viral load. However, we speculate that the parental virus with an unaffected primer binding site was vertically transmitted from the dam to its fetus through the placental barrier. This parental virus was still present in this organ, while the observed deletion mutant virus evolved in other parts of the fetus. The deletion found in the brain and unidentifiable organ sample was located in a genomic region which has been previously described as a mutation hot spot [14,28], and affected the M-segment based SBV real-time RT-PCR. In addition, this specific region of high sequence variability, specifically the amino terminal 234 amino acids (aa) of the envelope protein Gc, represents a major domain connected with the induction of virus neutralizing antibodies [16–18] and which was described before for other bunyaviruses as being not essential for in vitro growth [20]. Therefore, it was tempting to conclude that such deletions are related to immune evasion mechanisms within this region.

### M-segment variation in malformed fetuses is the consequence of targeted immune escape

**Fig 1:**
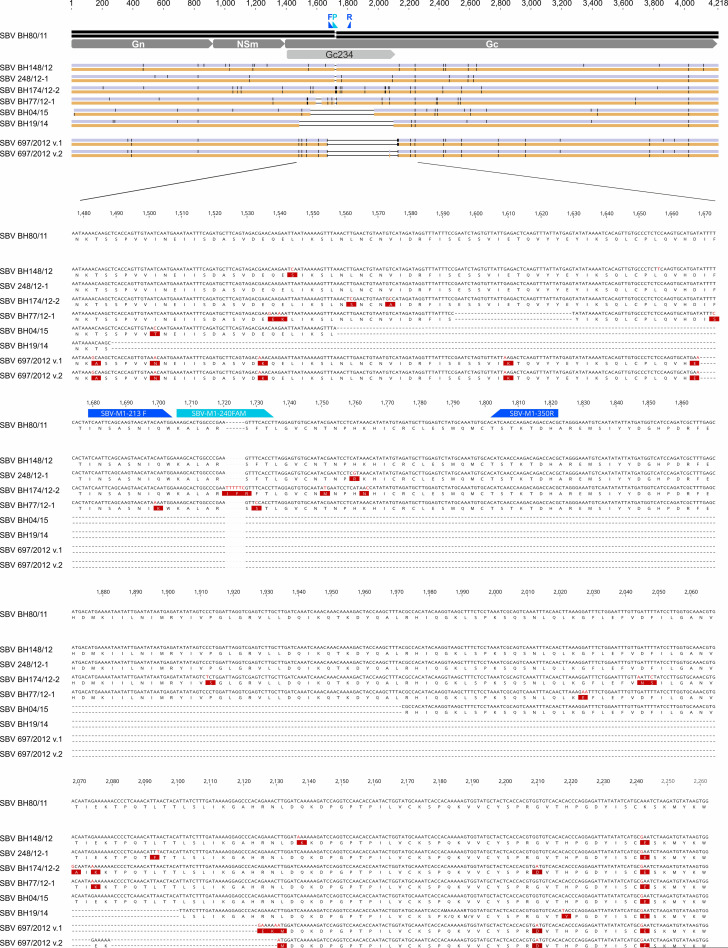
Comparison of the M-segment sequences of SBV strains used in this study. The first SBV (BH80/11), which was isolated in 2011, was used as reference strain. Nucleotide (purple bar alongside the name of each isolate in the upper panel) and amino acid (orange bar) substitutions are highlighted as vertical black lines (upper panel) or are highlighted in red (lower panels). The relevant section of the M-segment is magnified to display details, including the binding sites of the forward (F) primer, probe (P) and reverse primer (R) of the real-time RT-PCR in blue. For SBV strain 697/2012, two possible alignment version are shown.

In order to investigate the potential mechanisms further, the reactivity of SBV-specific neutralizing antibodies with various M-segments of SBV was analyzed. The first SBV isolate (BH80/11), which was initially obtained from a blood sample of a viremic cow in 2011 [1], was used as the reference strain. In addition, two strains with aa substitutions in the M-segment (BH148/12 and BH248/12-1) [14], one strain with an insertion of 2 aa (BH174/12-1) [14], another one with a deletion of 12 aa (BH77/12-1) [14], and a strain with a deletion of 150 aa (697/2012) in the N-terminal domain of Gc were included in this study (Fig 1). Furthermore, an SBV from 2014 (submission number BH19/14), which was detected in the liquor cerebrospinalis of an ovine fetus, was integrated in the present study. This sample tested positive by the S-segment based real-time PCR (Cq 27.2), and sequence analysis performed as described above revealed an even larger in-frame deletion in the Gc coding region (612 nt; Fig 1) than was observed in strain BH04/15.

**Fig 2:**
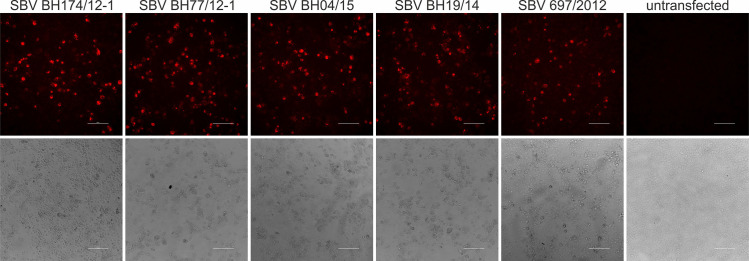
Immunofluorescence staining of SBV M-segments from viruses BH174/12-2, BH77/12-1, BH04/15, BH19/14, and 697/2012 with a polyclonal rabbit serum specific for SBV-Gc aa 890–1326. This staining was used to verify expression of the M-segments of all viruses which did not react with any of the applied Gc-specific mAbs.

Since it was not possible to isolate virus from the diagnostic samples BH04/15 and BH19/14 or to generate infectious virus containing the large deletions identified in these samples by a previously described reverse genetics system [29], BSR-T7/5 cells [30] that constitutively express the T7 RNA polymerase were transfected with cloned M-segment cDNA plasmids for all different SBV strains. The successful transfection of the M-segment plasmids was confirmed by staining with a polyclonal rabbit antiserum (Fig 2) generated against the conserved c-terminal region of SBV Gc (aa 890–1326).

**Fig 3:**
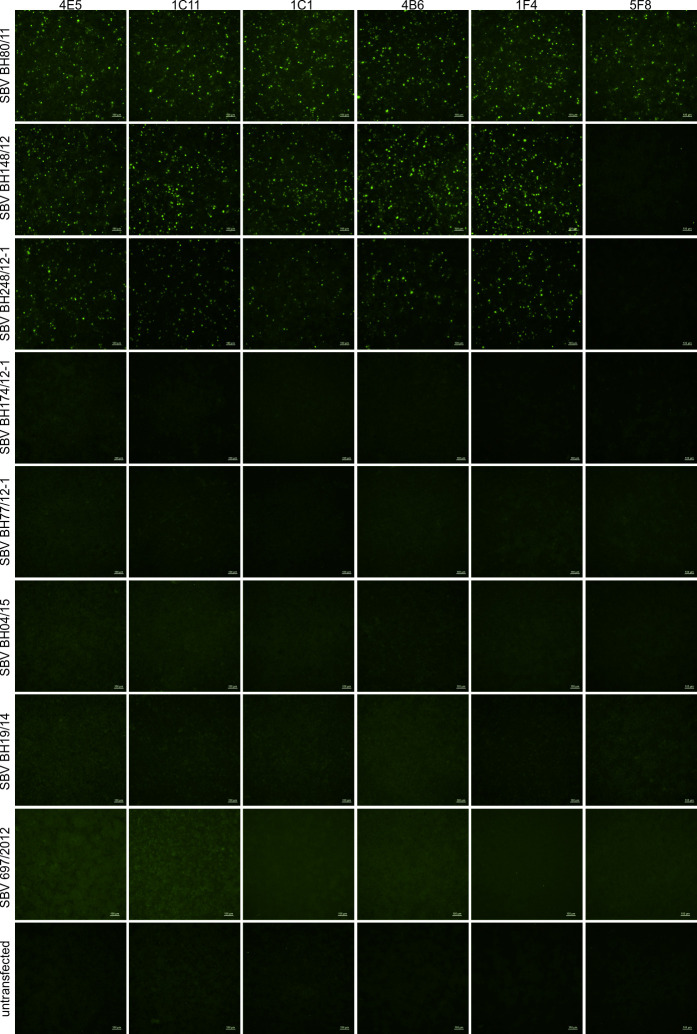
Immunofluorescence reaction pattern of SBV BH80/11, BH148/12, BH248/12-1, BH174/12-2, BH77/12-1, BH04/15, BH19/14, and 697/2012 stained with neutralizing SBV-specific monoclonal antibodies. The name of the respective SBV is shown on the left and the names of the antibodies are indicated above the corresponding picture rows. Scale bar indicates 100μm.

The original SBV isolate BH80/11 reacted with each of the tested SBV-specific anti-Gc monoclonal antibodies while the M-segment of the virus strains BH148/12 and BH248/12-1 which display only a few aa substitutions across the M-segment could be stained by five out of six monoclonal antibodies (Fig 3). The only exception was antibody 5F8 which has previously been shown to be specific for SBV strain BH80/11 [31]. However, none of the strains with aa insertions (BH174/12-1) or deletions (BH77/12-1, BH04/15, BH19/14, and 697/2012) within the Gc coding region reacted with any of the tested neutralizing anti-SBV monoclonal antibodies independent of the length of the respective insertion/deletion (Indel) (Fig 3).

**Fig 4:**
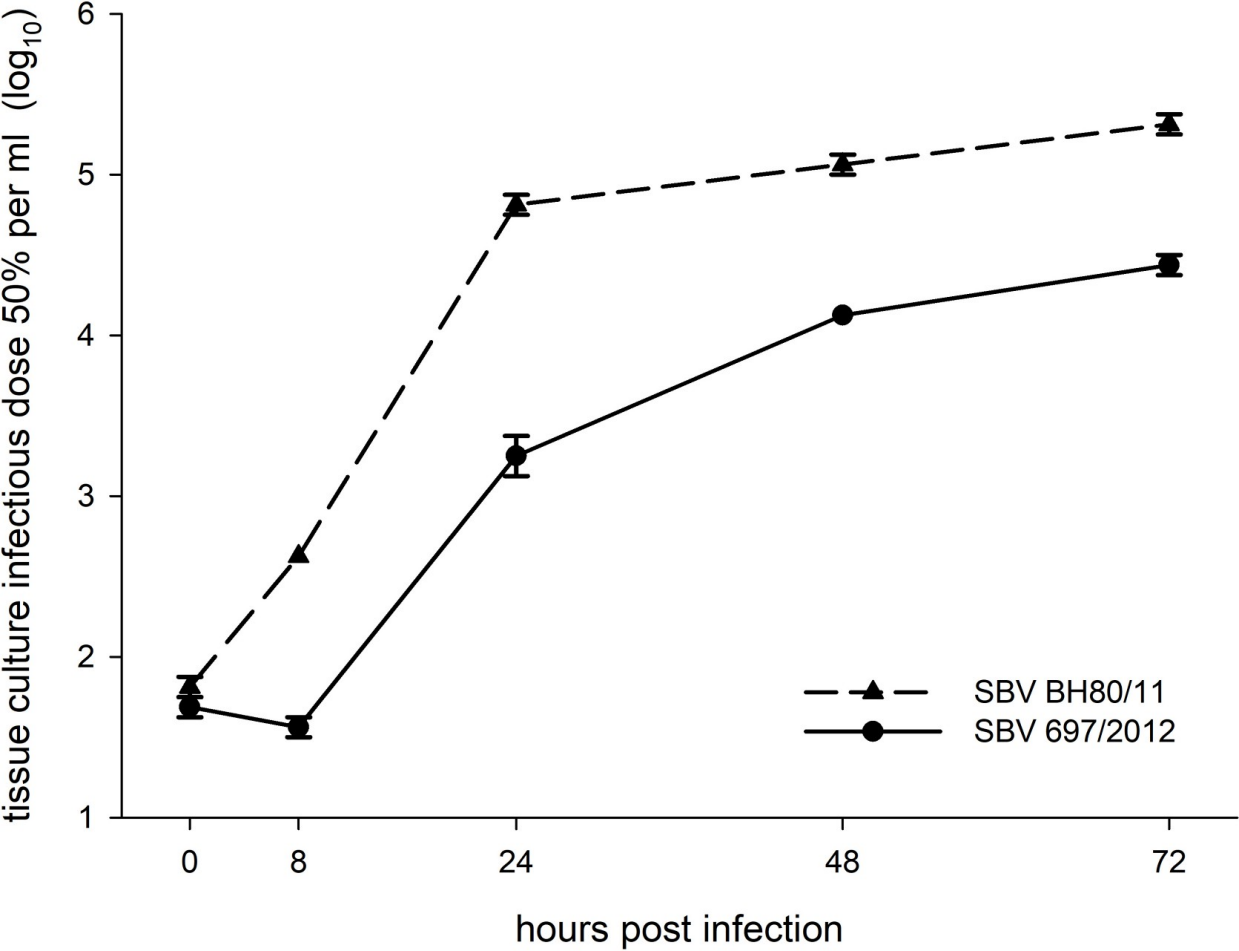
Growth kinetics of SBV isolates BH80/11 and 697/2012 in BHK cells. The cells were infected with a multiplicity of infection of 0.1; supernatants were collected at the times indicated in the figure, and titers were measured by endpoint titration in BHK cells. The experiments were performed in duplicates and mean values and standard deviations (error bars) are shown.

Despite all efforts to isolate virus from highly positive Indel virus containing samples or generate recombinant viruses using reverse genetics, only strain 697/2012 could be isolated on baby hamster kidney-21 (BHK-21) cells. Subsequently, the *in vitro* growth of this very unique virus isolate and the SBV reference strain BH80/11 were compared to each other; the strain 697/2012 showed impaired growth characteristics (Fig 4), a phenomenon already described for Bunyamwera orthobunyavirus mutants that contained N-terminal deletions in their Gc protein [20]. Furthermore, both SBV strains were titrated in 10-fold dilution steps starting from 10^4.75^ tissue culture infectious dose 50% per ml (TCID_50_/ml) on mammalian cells in the presence of either monoclonal anti-Gc antibodies [31], sera of experimentally infected cattle [32] or sheep [33] or sera of animals immunized with the recombinant amino terminal 234 aa of SBV Gc or a conjugation product of this domain with the corresponding genomic region of AKAV [34].

**Fig 5:**
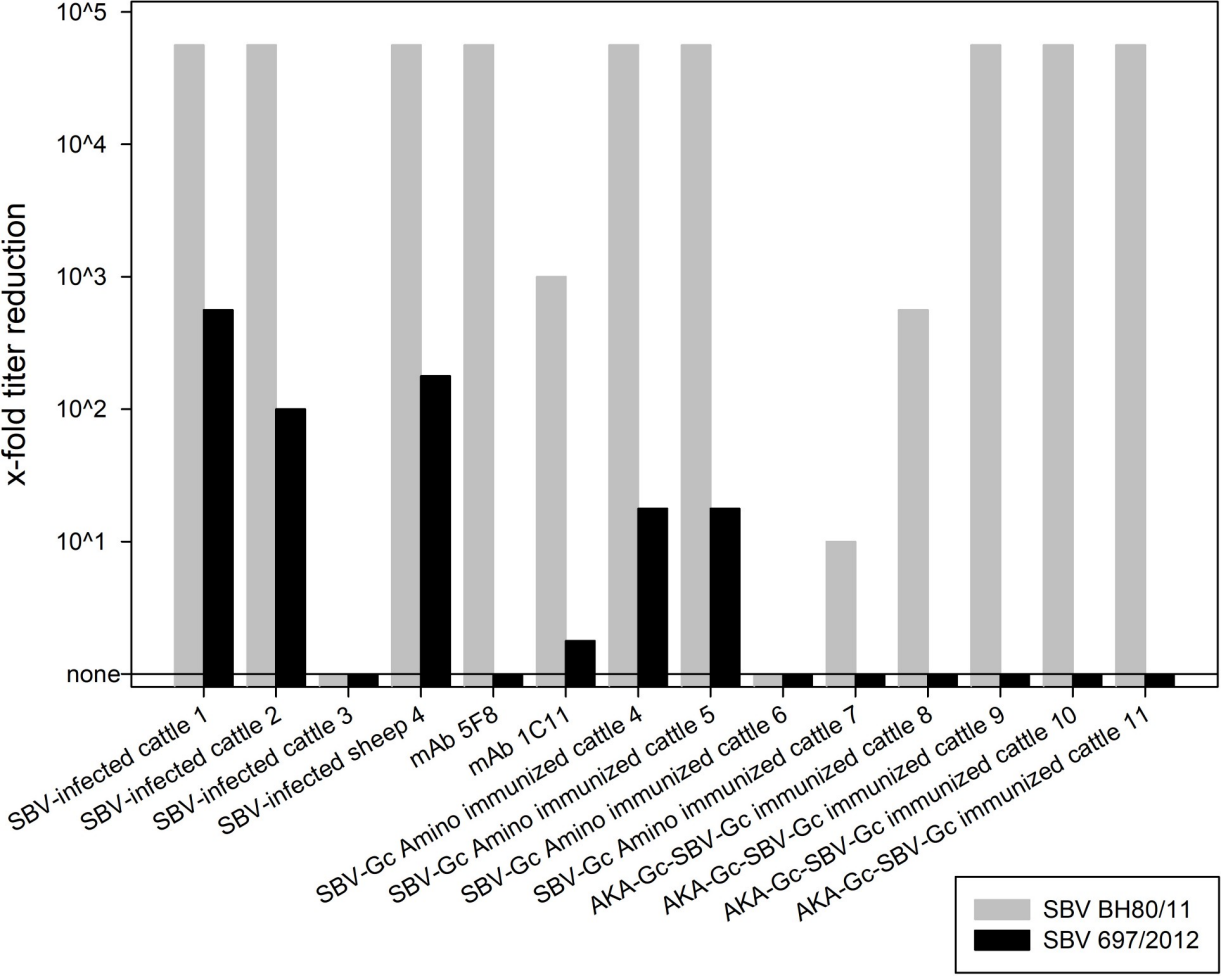
Reduction of virus titers of SBV isolates BH80/11 and 697/2012 grown for 72 hours in the presence of sera collected from cattle (cattle 1 to 3) and sheep (sheep 4) infected with SBV, cattle immunized with glycoprotein Gc subunit vaccines (cattle 4 to 11 [34]), or monoclonal anti-Gc antibodies (mab [31]). The titer reduction is calculated in relation to the final virus titers for the respective viruses grown in the presence of fetal calf serum. The neutralizing titers of the mabs and the cattle and sheep sera are given in parenthesis after the name of the respective mab or serum sample in the supporting information S1 Data.

As expected, the incubation of SBV strain BH80/11 with the aforementioned sera or anti-Gc antibodies resulted in a very efficient reduction or even a complete neutralization of virus growth in all cases (Fig 5 and S1 Data). In contrast, the M-segment deletion variant virus 697/2012 grew to titers of up to 10^2.75^ TCID_50_/ml despite the presence of sera from animals infected with wild-type SBV. The effect of anti-Gc antibodies was equally impaired, and the sera collected from cattle immunized with subunit vaccines had hardly any effect on the *in vitro* growth of this virus (Fig 5).

The envelope glycoproteins of orthobunyaviruses are targeted by the host humoral immune response [35], and the Gc represents the major immunogen targeted by neutralizing monoclonal antibodies [31,36–38]. The M-segment encoded Gc-protein of all mutated SBV strains tested in the present study showed a complete loss of the binding to a series of neutralizing antibodies against glycoprotein Gc [31]. This is in line with the observation that most of the aa exchanges and especially the large deletions are within the N-terminal Gc-domain which was recently characterized as the genome region responsible for SBV neutralization [16–18]. As a consequence of the fact that the SBV mutation hot spot is located in the Gc-coding region, the high sequence variability was most likely related to viral immune escape mechanisms. When naïve dams are infected with SBV during a vulnerable period of pregnancy the circulating virus may cross the placental barrier and infect the developing fetus. Antibodies that are produced by the fetus once it becomes immunocompetent at about 90 days of gestation [39] could assist in the clearance of the virus from the fetus since precolostral neutralizing antibodies are detectable in lambs or calves which test negative for the viral genome [13,40]. However, in a very high percentage of antibody-positive fetuses or newborns SBV RNA can also be detected [13,40,41]. Furthermore, exceptionally high viral loads together with high antibody levels can be found in malformed lambs or calves [12,13,40], suggesting that under the pressure of neutralizing antibodies, virus replication results in sequence adaptions within the antigenic domain of SBV. In the present study, the generation of escape mutants within the fetus is further demonstrated in samples from a lamb in which the parental virus seems to be still present in the lung, but extensive sequence alterations emerged in the virus variant detectable in further organs. The observed aa deletions in these samples and the sequence mutations in further SBV strains found in malformed fetuses are located within the previously described mutation hot spot within the M segment (aa 493 to 629) [14,28] which co-localizes with SBVs key immunogenic domain [16,18]. When used as a subunit vaccine this domain can even provide complete protection from virulent virus challenge [34]. The immune-dominance of the Gc protein is further reflected by the pattern of monoclonal anti-SBV antibodies arising in mice. Although the donor mouse was immunized with viral particles, no hybridomas secreting antibodies against Gn resulted [31].

In the present study, every neutralizing antibody from the recently characterized panel was used and when aa deletions were present within the mutation hot spot of the Gc coding region the resulting proteins did not react with any of the antibodies targeting the major neutralizing domain of SBV. Such regions of high sequence variability are a well-known phenomenon in viral proteins that play a role in the host-cell attachment and act as major immunogens involved in the induction of neutralizing antibodies [42–45]. Similar to the mutation accumulation in the envelope protein GP5 of porcine reproductive and respiratory syndrome virus [45], the haemagglutinin protein of influenza virus [42], or the E2/NS1 of hepatitis C virus [46], the hypervariable region of SBV seemingly plays the key role in the evasion of host immunity.

In the case of SBV, however, these escape variants are only observed in viruses arising in fetuses whose mothers were infected during the vulnerable period of pregnancy. The viral genome detected in the blood of acutely infected, viremic animals is highly stable, and all M-segment sequences generated from circulating SBV strains between 2011 and 2019 cluster very closely, independent of the year or European country of isolation [7,10,11,22].

The fact that SBV overcomes a second barrier besides the placental barrier, namely the blood-brain-barrier of the fetus, and that the virus is detectable in exceptionally high viral loads especially in the fetal CNS [12,14] may represent another escape mechanism. In adult animals, the highly selective semipermeable blood-brain barrier separates the circulating blood from the brain extracellular fluid. However, the brain endothelial cells and functionally effective tight junctions complete their development just before the time when immature ruminant fetuses become viable and, as a consequence, the fetal blood-brain barrier is permeable for higher-molecular weight compounds to a certain extent early in pregnancy [47,48]. Furthermore, specific fetal transport mechanisms across the blood-CNS barrier exist for plasma proteins which allow proteins to enter the CNS via the choroid plexuses to a greater extent than in the adult [49]. Thus, also antibodies can cross the developing blood-brain barrier [50,51].

The infection of fetuses and resulting malformations are complex events thus far poorly understood. From field observations it is known that a sizeable number of fetuses clear the virus upon infection leaving only antibodies as traces of previous infection, while other fetuses do not mount antibodies against the infection *in utero* [13]. Experimental infection of sheep with SBV demonstrates that infection of fetuses was overall difficult to reproduce at day 45 and day 60 [52], while in those cases where SBV is detected in newborn lambs a viral persistence in utero may be assumed [53]. This is in contrast to the very short viremia of SBV, where innate immune mechanisms seem to confer clearance of virus before antibodies become detectable. Taking into account also the experience from previous Akabane models [54] we hypothesize that SBV infections leading to fetal malformations occur relatively early during pregnancy (25–45 days in gestation in sheep). At this point in time the immune system of the fetus is still developing and not able to immediately clear the infection. This may be facilitated by the virus replicating in immune privileged organs, such as the brain. While the virus persists, it is recognized as danger by the immune system ultimately leading to an adaptive, antibody-driven immune response, which exerts pressure on the virus to escape, or allows to clear the virus from the fetus at this point. This model is in agreement with the above mentioned finding of viral persistence in fetuses [53], the observed mutations in the M-segment [14] as well as the detection of antibody positive, virus negative newborn lambs [41]. The most pertinent question arising for SBV was whether a resulting isolate would be perfectly adapted to replicating in sheep or would rather lose its pathogenicity due to the mutation occurring in the neutralizing domain of its receptor protein.

### Deletion in the Gc immunogenic domain leads to attenuation in target animals

In order to assess the pathogenicity of the unique SBV Gc deletion variant 697/2012, which could be isolated out of brain material of a malformed ovine fetus, six lambs were inoculated with the culture-grown virus. None of the animals showed clinical signs of an SBV infection, such as fever or diarrhea, and viral RNA could not be detected throughout the observation period of 14 days. In addition, serological assessment of samples taken from each animal on days 0, 7, 14, 21 and 28 after infection remained negative for SBV antibodies. The loss of infectivity in the *in vivo* model, which is in contrast to viruses with complete M-segments that induce viremia and seroconversion in ruminants [1,55,56], further indicates that SBV M-segment variant viruses are a natural artefact, emerging only in infected fetuses and cannot be transmitted further. However, it is important to mention that they are not just “defective interfering particles (DIs)” which could occur in the presence of wildtype SBV, since (i) wildtype virus has not been isolated from these cases and highly sensitive PCR-analysis targeting the deleted region is negative in brain material from those cases, (ii) (deep) sequencing does not show fragments encoding the deleted M-segment parts, and (iii) the here characterized isolate replicates efficiently *in vitro* without any help by a wildtype SBV. Overall, it seems that special conditions–like in the fetal brain–support development and growth of such SBV mutants. Isolation with standard cell culture methods, however, seems to be less successful.

Concordantly, those variant viruses have never been observed as circulating viruses in acutely infected adult animals. As pointed out earlier, the viral genome detected in acutely infected, viremic animals is more stable and does not show any of the insertions or deletions [11,22]. Moreover, insect-transmitted viruses such as SBV have to adapt to two different hosts, namely the arthropod vector and the mammalian host, and undergo replication cycles in both hosts. The high sequence stability might be necessary for the transmission between hosts as it has been described for other vector borne viruses like West-Nile virus [57–59]. SBV variants as we describe here arising in malformed fetuses result in an evolutionary *cul de sac* where transmission becomes impossible.

## Conclusions

We suppose that the observed variation of the M-segment of SBV present in malformed fetuses is most likely the consequence of a specific immune escape within the CNS of chronically infected malformed fetuses. These variant viruses are a kind of artefact and exclusively generated due to this unique combination of high replication rates of SBV in the fetal CNS and the parallel immune pressure of neutralizing antibodies. Developing fetal brain tissues have e.g. different immunity patterns presumably allowing replication of these mutants. Furthermore, restriction to mainly brain tissues is limiting accessibility for the insect vector thereby drastically reducing the chance for transmission. It is therefore very unlikely that variant viruses enter the usual insect-mammalian host cycle since they are not transmitted to adult animals and thus, do not cause viremia. Hence, we hypothesize that the different SBV M-segment mutants are always newly generated in the infected fetus. This also explains the high number of different variants with numerous combinations of mutations resulting in the same escape from neutralization.

Consequently, such mutant orthobunyavirus strains from fetal brain tissues are not representing circulating strains and should not be included in attempts to trace the spatial-temporal evolution of orthobunyaviruses in molecular-epidemiological approaches during outbreak investigations.

## Materials and methods

### Ethics statement

The sheep experiment was conducted within animal housing facilities at the APHA following Home Office guidelines in accordance with PPL 70/7503.

### Diagnostic samples and SBV strains

In February 2014, the liquor cerebrospinalis sample of a malformed ovine fetus (submission number BH19/14), and in January 2015, different tissue samples (brain, lung, placenta, two unspecified organ samples) of a malformed lamb (submission number BH04/15) were submitted to the Friedrich-Loeffler-Institut to be analyzed for the presence of SBV-genome.

In addition, the first SBV isolate (BH80/11 [1]), which was used as the reference strain, two strains with aa substitutions in the M-segment (BH148/12 and BH248/12-1), one strain with an insertion of 2 aa (BH174/12-1), a strain with a deletion of 12 aa (BH77/12-1) [14], and a strain with a deletion of 150 aa (697/2012) in the N-terminal domain of Gc were included in this study. The strain 697/2012 was newly sequenced by Sanger sequencing performed in both directions using the Big Dye Terminator Mix (Applied Biosystems, Darmstadt, Germany) with the correlating tagged primers on the ABI 3730 sequencer as described [60]. The sequences were submitted to DDBJ under accession numbers LC557487 (S-segment), LC557488 (M-segment) and LC557489 (L-segment). When comparing this sequence to the first SBV isolate from 2011 (BH80/11), two alignment versions appear, either a large in frame deletion of 150 aa combined with 4 nt substitutions at the 3’ end of the deletion, or a discontinuous deletion (Fig 1). The sequences of the remaining aforementioned SBV strains have been previously described and were obtained from NCBI GenBank.

### Extraction of nucleic acid, real-time RT-PCR, and sequencing

Viral RNA was extracted from the diagnostic samples using the QIAamp Viral RNA Mini Kit (Qiagen GmbH, Hilden, Germany) according to the manufacturer’s recommendation and analyzed for the presence of SBV-genome by independent real-time RT-PCR assays based either on the S-, the M-, or the L-segment [12,23]. Sequencing of the M-segment of SBV strains BH19/14 and BH04/15 was performed in both directions by termination cycle sequencing using the Big Dye Terminator Mix (Applied Biosystems, Darmstadt, Germany) on an ABI 3130 Genetic Analyzer (Applied Biosystems, Darmstadt, Germany) as described previously [14]. Sequence alignments and translation in amino acids (aa) were performed using Geneious version 10.0.9 (Biomatters, Auckland, New Zealand).

### Transfection with cloned M-segment cDNA plasmids

The M-segment plasmids with mutations, deletions and insertions in the hypervariable region according to the wild-type sequences of strains BH148/12, BH248/12-1, BH174/12-1, BH77/12-1, BH04/15, BH19/14 and 697/2012 were generated by fusion PCR on the basis of the cDNA construct pT7ribo_SBV_M which displays the SBV strain BH80/11 sequence downstream of a T7 promoter [29]. Primer sequences and further information regarding the cloning details are available upon request. All plasmids were verified by DNA sequencing using the BigDye Terminator v1.1 Cycle Seq. Kit (Applied Biosystems, Darmstadt, Germany). Plasmid DNA was purified by Qiagen Plasmid Mini or Midi Kits according to the manufacturer’s instructions. Semi confluent layers of BSR-T7/5 cells seeded in 48-well microplates were transfected with 1.25 μg of the respective plasmid DNA using SuperFect transfection reagent (Qiagen, Hilden, Germany) according to the manufacturer’s protocol and incubated for three days at 37°C. Thereafter, cells were fixed for 2h at 80°C and stained by neutralizing monoclonal anti-Gc antibodies [31] or a polyclonal rabbit antiserum directed against amino acids 890 to 1326 of the SBV Gc ectodomain. As secondary antibodies, a fluorescein isothiocyanate (FITC-) conjugated goat anti-mouse IgG (Sigma-Aldrich Co., Steinheim, Germany) or a Alexa Fluor 594-conjugated F(ab')2-goat anti-rabbit IgG (Invitrogen, Darmstadt, Germany) were used, respectively. All antibody dilutions were prepared in Tris-buffered saline with 0.1% Tween-20 (TBST).

### Virus isolation, growth kinetics, and titer reduction assays

The samples of the diagnostic submissions BH 19/14 and 04/15 were homogenized in phosphate-buffered saline (PBS) and inoculated onto *Culicoides variipennis* larvae (KC) and baby hamster kidney (BHK) cells as described previously [1] or directly onto BHK cells.

Isolation of the ovine SBV strain 697/2012 was carried out from brain material of a neonate sheep with severe malformation, delivered dead at parturition. Approximately 2g of brain was homogenized in PBS. 300μl supernatant of this homogenate was inoculated onto a sub-confluent monolayer of BHK cells. Following an incubation of 3 hours at 37°C/5% CO_2_ the inoculum was removed and replaced with Minimum Essential Medium (MEM) supplemented with 10% fetal calf serum (FCS). After two cell passages a cytopathic effect (CPE) was visible and the presence of SBV was confirmed by real-time RT-PCR and sequencing of the S segment.

*In vitro* growth kinetic experiments were performed using BHK cells which were inoculated with either SBV isolate 697/2012 or BH80/11 with a multiplicity of infection (MOI) of 0.1; experiments were performed in duplicates. Supernatants were collected at 0, 8, 24, 48, and 72 hours post infection and titers were calculated by counting CPE-positive wells of BHK cells and displayed as TCID_50_/ml.

10-fold dilution series of the SBV reference strain BH80/11 and the newly isolated Gc deletion variant 697/2010 were prepared in MEM starting with a titer of 10^4.75^ TCID50/ml as determined by endpoint titration using BHK cells. The medium was supplemented with 10% of either FCS (negative control), monoclonal anti-Gc antibodies [31], sera of cattle [32] or sheep [33] experimentally infected with SBV or sera of animals vaccinated with the amino terminal 234 aa of the Gc protein of SBV alone or in combination with the corresponding genomic region of AKAV [34]. The dilution series were incubated for 3 days at 37°C/5% CO_2_ on BHK cells, and subsequently assessed for CPE. The titre reduction was calculated in relation to the final virus titres of the respective virus grown in the presence of FCS.

### Inoculation of sheep

Six Cheviot lambs (<1 year old) were inoculated subcutaneously with 8x10^5^ plaque forming units (PFU) in 1ml of SBV isolate 697/2012. The animals were monitored daily to assess clinical disease. Whole blood samples were taken on day 0 and each day thereafter for a period of 14 days. Viral RNA was extracted as described above and analyzed by an S-segment based real-time RT-PCR [12]. Sera were collected on days 0, 7, 14, 21 and 28 and tested by a plaque reduction neutralization test (PRNT) [61]. Following the 28 day period lambs were terminated humanely.

## Supporting information

S1 Data Excel spreadsheet containing, in separate sheets, the underlying numerical data for Figs 4 and 5. (XLSX)
